# Trait gradients inform predictions of seagrass meadows changes to future warming

**DOI:** 10.1038/s41598-021-97611-x

**Published:** 2021-09-13

**Authors:** Arianna Pansini, Gabriella La Manna, Federico Pinna, Patrizia Stipcich, Giulia Ceccherelli

**Affiliations:** 1 Dipartimento di Architettura, Design, Urbanistica, Università di Sassari, Via Piandanna 4, 07100 Sassari, Italy; 2 MareTerra Onlus-Environmental Research and Conservation, Regione Sa Londra 9, 07041 Alghero, Italy; 3 Dipartimento di Chimica e Farmacia, Università di Sassari, Via Piandanna 4, 07100 Sassari, Italy

**Keywords:** Climate-change ecology, Heat

## Abstract

Comparing populations across temperature gradients can inform how global warming will impact the structure and function of ecosystems. Shoot density, morphometry and productivity of the seagrass *Posidonia oceanica* to temperature variation was quantified at eight locations in Sardinia (western Mediterranean Sea) along a natural sea surface temperature (SST) gradient. The locations are spanned for a narrow range of latitude (1.5°), allowing the minimization of the effect of eventual photoperiod variability. Mean SST predicted *P. oceanica* meadow structure, with increased temperature correlated with higher shoot density, but lower leaf and rhizome width, and rhizome biomass. Chlorophyll *a* (Chl-*a*) strongly impacted seagrass traits independent of SST. Disentangling the effects of SST and Chl-*a* on seagrass meadow shoot density revealed that they work independently, but in the same direction with potential synergism. Space-for-time substitution predicts that global warming will trigger denser seagrass meadows with slender shoots, fewer leaves, and strongly impact seagrass ecosystem. Future investigations should evaluate if global warming will erode the ecosystem services provided by seagrass meadows.

## Introduction

Global warming is expected to have profound consequences on biodiversity and functioning of major systems on Earth^1,2^. The impact of temperature increase has been measured over the past two decades^3–6^, but understanding how this physical forcing affects ecosystems is unclear, particularly in the sea^7–9^. This, however, is critical for predicting the consequences of global warming and identifying mitigation and restoration actions.

Much of experimental temperate marine coastal ecology is focused on elucidating how temperature increases will impact the physiology, fitness and distribution of organisms. Two main approaches are being employed to examine warming effects: (I) experiments with artificial heating such as mesocosms^10–13^ and (II) monitoring the response of organisms to temporal or spatial variation in temperature, across years^14–16^ or latitude^17–20^. Each of these approaches has advantages and drawbacks. Manipulative experiments may examine responses to temperature or patterns not yet under natural conditions, such as intense, long lasting heat waves^21–23^. Experiments are typically done at small spatiotemporal scales and often ignore covarying abiotic conditions including light availability^24^, UV irradiation and acidification^25,26^, or biotic effects such as predation^27,28^. Conversely, comparing populations across sites with varying temperatures, such as latitudinal gradients, can provide information about the role of warming on the structure and function of future ecosystems, but it is often difficult to disentangle temperature from other covarying effects, such as photoperiod, light quality and quantity^29^. Moreover, marine sea surface temperature (SST) is commonly linked to chlorophyll-a (Chl-a), with high-temperature locations having low-nutrient availability and Chl-a^30,31^ and high light attenuation^32,33^. Problems between laboratory and field results are not surprising, since temperature, nutrients and irradiance effects may be cumulative or antagonistic depending on the species and system.

Therefore, uncertainties with warming effects on marine biota are also indirectly due to co-variation between SST and Chl-*a*. While there are latitudes where these patterns are predictable, regional anomalies are also found especially where upwelling occurs^34^. Nevertheless, SST increase does not necessarily imply decreasing Chl-*a*, suggesting that complex processes, such as advection, define sea water conditions^34^. Further variability of marine species response to warming comes from natural variation in physiological, morphological and life-history attributes (functional traits) among populations, as there is evidence of adaptation to spatial temperature gradients in many organisms and at different scales^35–39^. Species phenotypic gradients presumably can reflect patterns of genetic differentiation and local adaptation, making additional data potentially necessary to estimate how much of observed phenotypic differences are due to plastic responses versus adaptive differentiation between populations.

Understanding future warming effects on foundation species, as marine macrophytes, is pivotal to predict their distribution and physical structure^40^, as temperature is thought the most important range limiting factor^41^. Seagrasses are valuable providers of coastal ecosystem services including, carbon sinks, nursery grounds, habitat, nutrient cycling, sediment stabilization, trophic transfer to adjacent habitats^42–44^ and protection from erosion^45,46^. *Posidonia oceanica* (L.) Delile is a slow-growing seagrass, endemic to the Mediterranean, experiencing widespread decline due to multiple local anthropogenic stressors^47^. The abrupt decline experienced by *P. oceanica* from recent heatwaves^48^, however, has seriously questioned its persistence for the coming decades^40^. Due to its vulnerability in aquaria and slow growth, laboratory experiments have been limited and controversial. Nevertheless, plants from warm thermal environments were found to activate a suite of physiological^49^ and molecular mechanisms^50–52^ to tolerate simulated heatwave exposures, whereas phenological response to warming likely involves higher flowering^53^ and denser meadows^54^.

This is a space-for-time substitution, a method for studying slow ecological processes, where the relationships between ecological variables are studied at sites that are assumed to be at different stages of development^55^. This study is based on the assumption that plant functional traits vary along environmental gradients and potentially predict responses to environmental change. Thus, to examine the performance of *P. oceanica* to future temperature conditions, we measured shoot density, morphometry and productivity at eight locations in Sardinia (western Mediterranean Sea) along a natural gradient of water temperature. Despite similar latitude (minimum interference of photoperiod), the western locations are generally cooler than the eastern sites, with differences in SST comparable to climate change scenarios for the twenty-first century for the Mediterranean Sea (peaking at 2.6 °C in 2100^56,57^), making this space-for-time substitution informative for projections of trait changes over the next decades. Chl-a, a proxy of light irradiance, was a further driver of seagrass structure. *P. oceanica* is currently in the EU Marine Strategy Framework Directive monitoring protocols^58^.

## Results

### Seagrass variability

**Figure 1:**
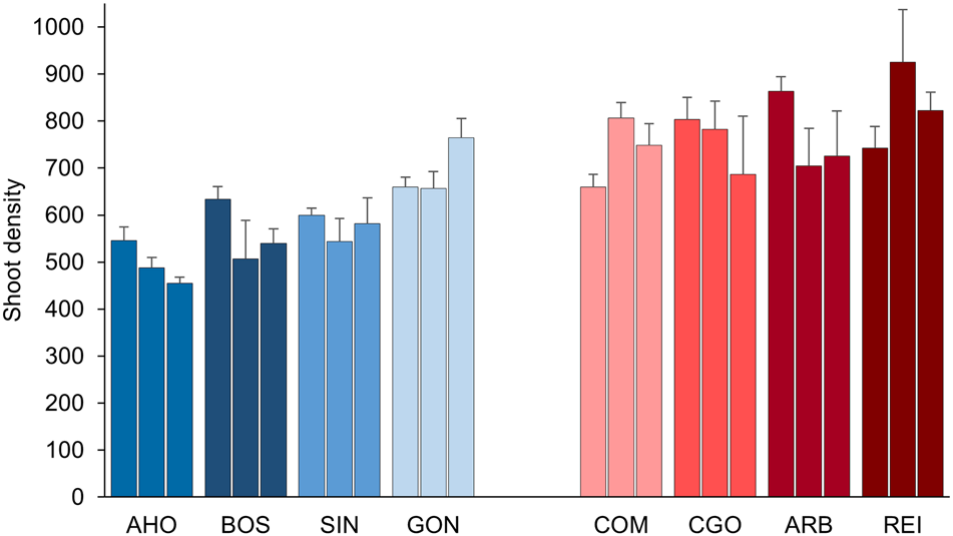
*Posidonia oceanica* mean (+ SE) shoot density (# of shoots/m^2^) at each location: in blue the western and in red the eastern. For each location data of the three areas are shown (n = 4).

**Figure 2:**
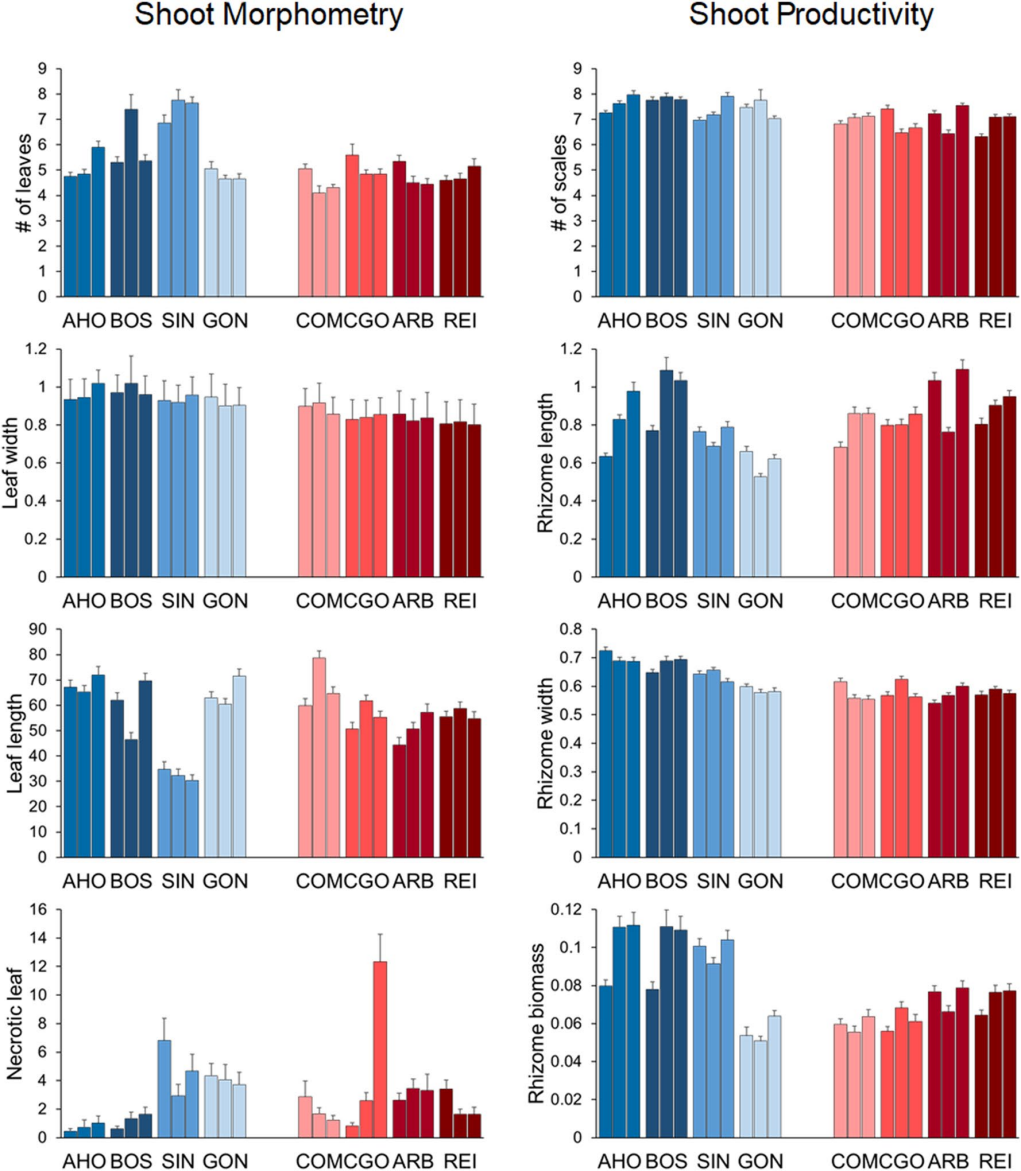
*Posidonia oceanica.* Mean (+ SE) morphometry (left) and productivity (right) variables. Morphometry: # of leaves/shoot, leaf width (cm), length (cm), and necrotic leaf portion (%). Productivity: # of scales/shoot*year, rhizome elongation (cm/year), rhizome width (cm/year) and rhizome biomass (g/year) across locations, in blue the western and in red the eastern. For each location data of the three areas are shown (n = 20).

Shoot density changed considerably between Sardinian coasts (Table 1 and Fig. 1) as well as leaf width which was larger on the west than on the east side (Table 1 and Fig. 2), although both variables were significant across locations and areas. All other morphometrical variables were significantly affected by location and area, except for necrotic leaf portion that was only dependent on the area (Table 1 and Fig. 2).

**Table 1:** ANOVAs: effects of coast (west vs east), location (4 levels), and area (3 levels) on *Posidonia oceanica* shoot density, morphometry (# of leaves, leaf width, leaf length, necrotic leaf) and productivity (# of scales, rhizome length) variables. For each ANOVA, the *F* values are given. In bold are the significant values (* for *p*-value significance level 0.01, ** for *p*-value significance level 0.001).

	Coast = C	Location (C) = L	Area (L(C))
	*F*_1,6_	*F*_6,16_	*F*_16,72_
**Shoot density**	14.37**	4.37**	1.89**
**Shoot morphometry**	*F*_1,6_	*F*_6,16_	*F*_16,72_
# of leaves	3.15	5.88**	5.32**
Leaf width	20.96**	4.18*	3.21**
Leaf length	0.01	11.82**	5.22**
Necrotic leaf	0.04	1.95	6.70**
**Shoot productivity**	*F*_1,6_	*F*_6,16_	*F*_16,456_
# of scales	20.45**	0.54	4.00**
Rhizome length	0.43	2.7	4.32**
Rhizome width	9.77*	5.56**	1.3
Rhizome biomass	5.3	4.20**	2.40**

The reconstruction analysis showed that annual plant productivity changed between coasts only in terms of number of scales (remnant leaf sheats) and rhizome width, being lower on the east coast. Rhizome width and biomass were significantly dependent on the location, while all other variables were highly area dependent (Table 1 and Fig. 2).

### Relationship between seagrass and environmental variables

**Figure 3:**
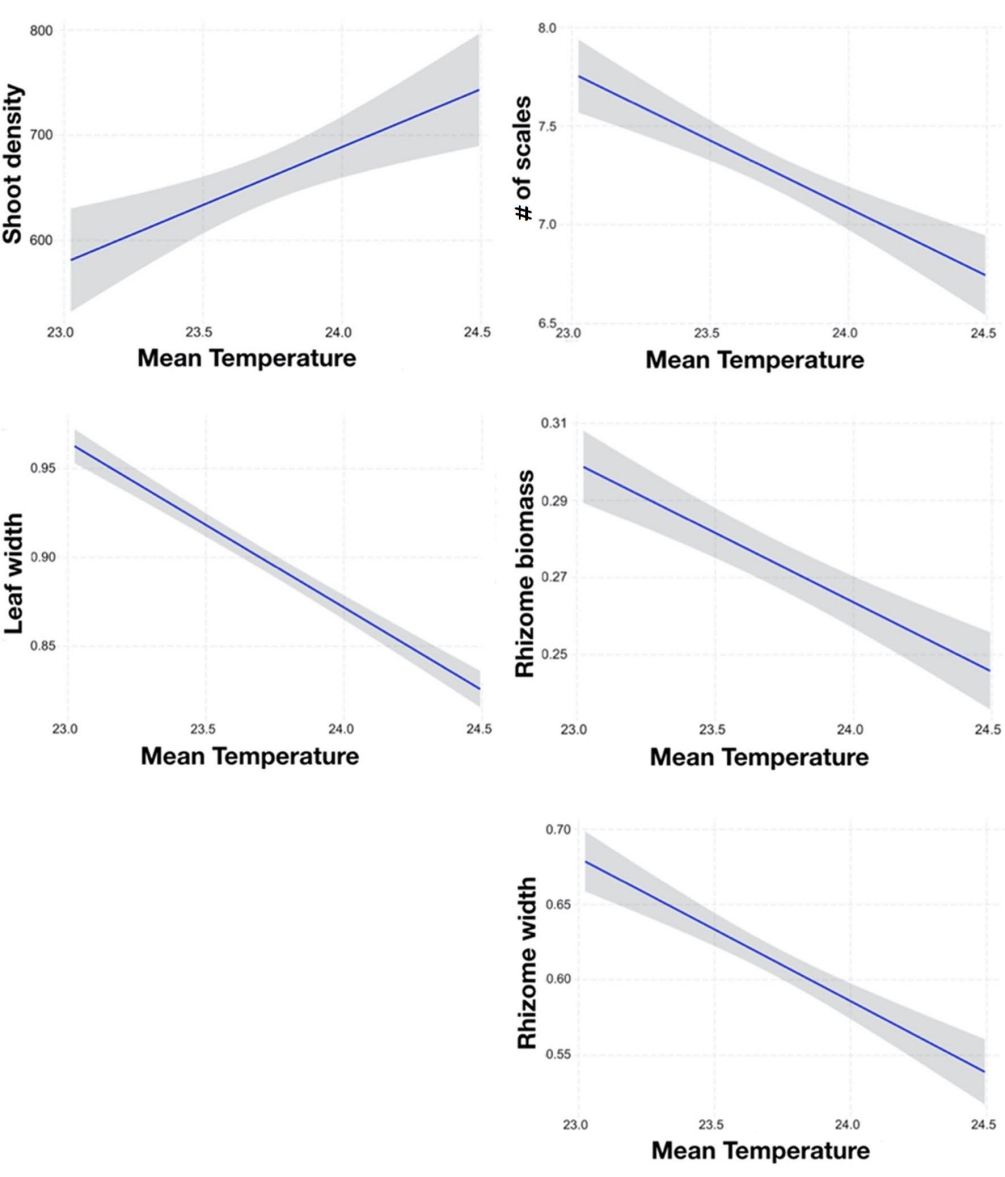
Plots from the multiple regression model of *Posidonia oceanica* response variables vs. mean temperature (°C).

**Figure 4:**
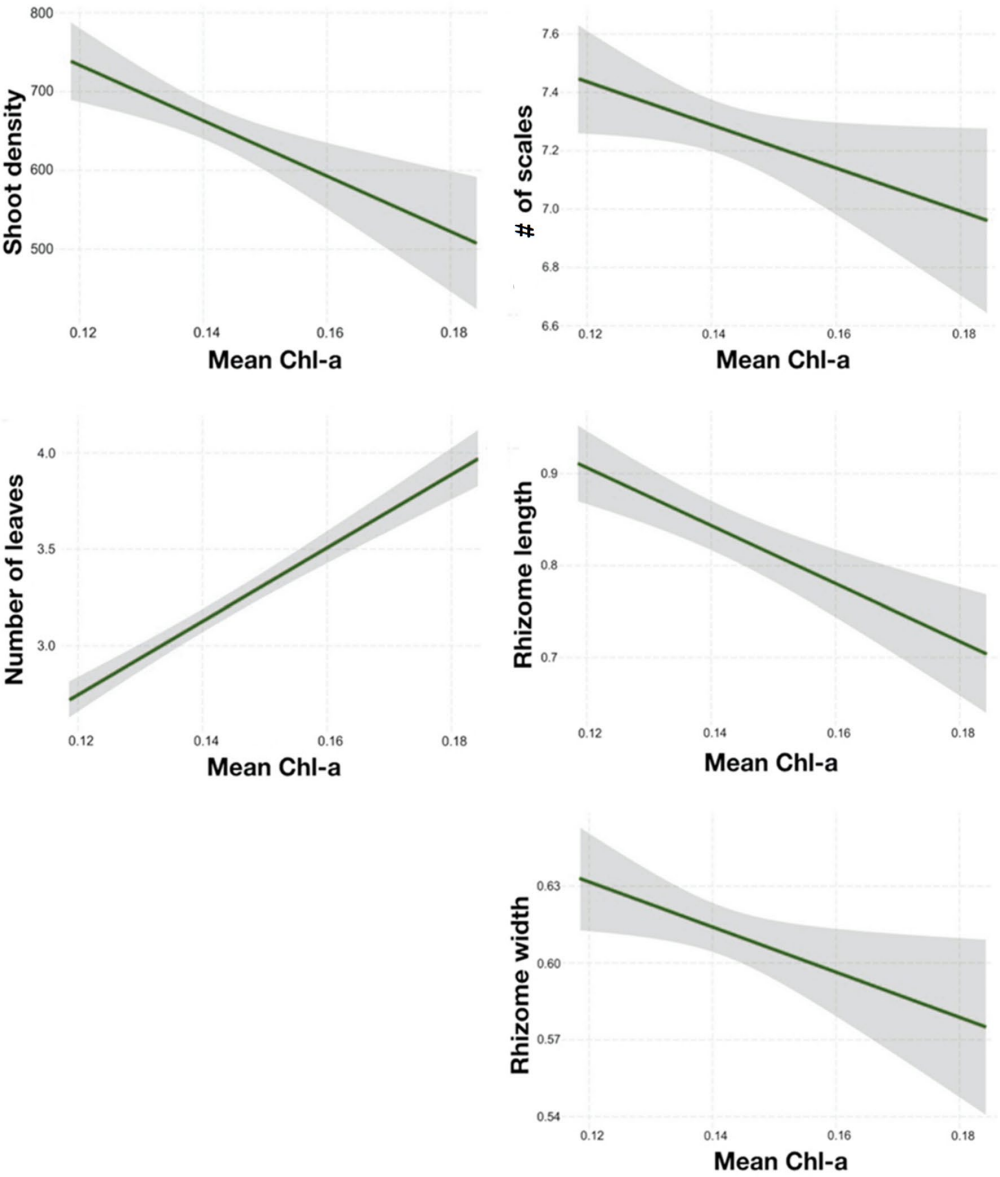
Plots from the multiple regression model of *Posidonia oceanica* response variables vs. mean Chl-*a* (mg/m^3^).

Multiple regressions retained only mean temperature in four models indicating that leaf width, number of scales, rhizome width and rhizome biomass were negatively related with mean temperature. Shoot density was related to mean temperature and Chl-a, as well as the number of scales and rhizome width (Table 2, Figs. 3 and 4). Specifically: I) increased shoot density was correlated with increased mean temperature, while an opposite trend was found for the leaf width, number of scales and rhizome width (Fig. 3) and II) reduced shoot density, number of scales and rhizome width were correlated with increased Chl-a (Fig. 4). The response variables where models retained Chl-a as the only explanatory variable, were the number of leaves and rhizome length, which increased and decreased, respectively, with increasing Chl-a (Table 2, Figs. 3 and 4).

**Table 2:** Multiple regressions outputs of *Posidonia oceanica* response variables against the predictors retained in the model by the backward selection.

Effect		Estimate	SE	t-value	p-value
**Shoot density**					
Mean temperature		110.22	31.31	3.52	< 0.001
Mean Chl-*a*		−3515.66	951.43	−3.69	< 0.001
Adjusted R-squared: 0.5422 F2,93: 57.26 p-value < 0.0001					
**Shoot morphometry**					
**# of leaves**					
Mean Chl-*a*		19.0687	1.578	12.08	< 0.001
Adjusted R-squared: 0.2324 F1,478: 146 p-value < 0.0001					
**Leaf width**					
Mean temperature		−0.093112	0.00533	−17.46	< 0.0001
Adjusted R-squared: 0.3925 F1,474: 305 p-value < 0.0001					
**Shoot productivity**					
**# of scales**					
Mean temperature		−0.6862	0.1191	−5.76	< 0.001
Mean Chl-*a*		−7.3927	3.6191	−2.04	0.0416
Adjusted R-squared: 0.0870 F1,476: 23.78 p-value < 0.0001					
**Rhizome length**					
Mean Chl-*a*		−3.15176	0.69724	−4.52	< 0.0001
Adjusted R-squared: 0.0393 F1,474: 20.43 p-value < 0.0001					
**Rhizome width**					
Mean temperature		−0.09516	0.01288	−7.39	< 0.0001
Mean Chl-a		−0.8811	0.39126	−2.25	0.0248
Adjusted R-squared: 0.1466 F1,477: 42.15 p-value < 0.0001					
**Rhizome biomass (sqrt)**					
Mean temperature		−0.035997	0.005231	−6.88	< 0.0001
Adjusted R-squared: 0.0880 F1,478: 47.35 p-value < 0.0001

Finally, the regression model indicated that shoot density was negatively related to the number of leaves and leaf width (Table 3).

**Table 3:** Multiple regression output of *Posidonia oceanica* shoot density against the predictors retained in the model by the backward selection.

Effect	Estimate	SE	t-value	p-value
**Shoot density**				
# of leaves	−114.24	39.27	−2.91	0.008
Leaf width	−1306.1	319.8	−4.08	< 0.001
Adjusted R-squared: 0.6762 F2,21: 25.02 p-value < 0.0001				

## Discussion

*Posidonia oceanica* morphometry and productivity were linked to the thermal environment. Increased temperature triggered higher shoot density, but lower leaf and rhizome width, fewer scales and lower rhizome biomass. Additionally, Chl-*a* was a temperature independent driver of the plant performance. Temperature strikingly affected shoot density, increasing gradually across the thermal gradient from 496.1 ± 21.6 to 829.9 ± 43.2 shoots/m^2^ (mean ± SE n = 12) at AHO and REI, respectively. Shoot density is the most common descriptor of *P. oceanica* meadows defining its conservation status (Marine Strategy Framework Directive) assuming that higher densities reflect lower human influence and better marine water conditions. However, the density classes distinguished by previous authors (reviewed by^59^), ignore natural environmental variation. Since our data were collected unaffected from local anthropogenic disturbances, our results highlight that thermal environment is critical factor in determining plant shoot density, providing evidence of the need to refer the seagrass density classes to the mean temperature environment.

**Figure 5:**
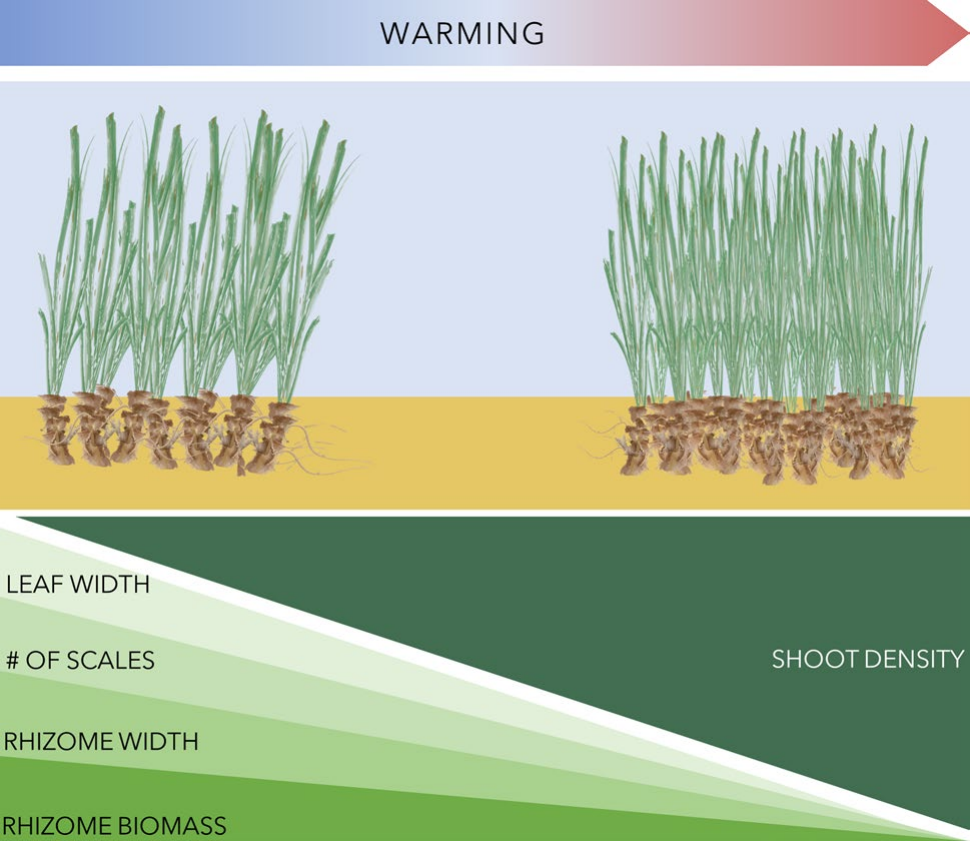
Summary of the results. Shoot density, morphometry and productivity was measured to examine the performance of the seagrass to the thermal gradient. Warming will trigger denser seagrass meadows with slender shoots (lower rhizome width and biomass) and fewer leaves (scales).

Our results revealed a strong spatial association between plant traits and temperature across a gradient suggest that future warming is predicted to produce denser *P. oceanica* meadows. This finding is corroborated by long-term correlative data revealing that shoot density is a plant trait that varies with thermal environment^54^, providing evidence that the plant would rearrange (increasing the number of modules) the meadows structure with warming (Fig. 5). The fact that Chl-*a* is inversely related to the meadow density will sharpen this pattern, as this influence is disentangled from temperature effects and because both drivers work in the same direction, enhancing shoot density and potentially producing synergistic effects. In fact, numerical models of future Chl-*a* due to anthropogenic climate change, generally suggest a decrease in globally integrated primary productivity driven by a reduction in supply of macronutrients^60–63^. Nevertheless, predicting meadow structure based on the relationship between spatial pattern of plant traits and the environment assumes that the seagrass traits could change proportionally to climate change^54^, although the species may respond to finer-scale changes in environmental variables that cannot be predicted using averages^64,65^.

Regarding mechanisms regulating the Chl-*a*-shoot density interaction, our data support the hypothesis that different light conditions due to the phytoplankton density (not nutrient availability) are involved, although manipulative experiments are needed. In fact, evidence of reduction of *P. oceanica* shoot density with depth are commonly gained^66–68^, supporting the hypothesis that light extinction is pivotal^69^. observed that seagrasses growing in low light reduce shoot density and above-ground biomass as an acclimation response to reduce self-shading within the canopy.

Shoot density changes induced by the climate change, however, will involve other phenological traits, such as leaf width and number of leaves. Their dependence on shoot density has been interpreted as the result of self-organization to shading^70–74^. Reducing the size of ramets to attenuate intraspecific competition is a common pattern in clonal plants^73,74^. Productivity of *P. oceanica* was not directly dependent on shoot density, but it seems that it will be contrastingly affected by the temperature and Chl-*a*, so that predicting the number of scales and rhizome width in coming decades is not obvious and likely dependent on the strength of their associations. Therefore, the prediction about the productivity that can be made on the trait gradients (trait variation along environmental gradient) regards the decrease in rhizome biomass and length affecting the plant robustness through decades.

Future changes in temperature and Chl-*a*, may drive *P. oceanica* morphometry and productivity patterns that will affect the ecosystem services that seagrass meadows currently provide. Quantification of seagrass services, however, have never been provided on a structure-specific basis^75,76^ and we believe this might become a relevant issue. Indeed, in a future warmer Mediterranean Sea, where summer mean SST increase will likely peak 2.9 °C and 2.7 °C for the end of the century on the east and west Sardinia coasts, respectively^56^, *P. oceanica* leaf canopy, structured by higher shoot density with bundles of a lower number of leaves smaller in width, can create a different habitat and associated community. Similarly, whether the reduction in rhizome width and biomass has consequences on both the vulnerability of plants to storms and Carbon storage remains unanswered.

This study shed light on how seagrass systems could respond to climate change, independently of the effects of extreme events (such as heat waves), as the latter undoubtedly affect deleteriously the seagrass structure with die-offs^47,77,78^. Nevertheless, the extent the phenotypic gradients of the seagrass systems depend on acclimation versus adaptation processes should be measured. However, the analysis of processes involved in phenotypic plasticity and the possibility that such plastic responses might be adaptive is complex for both the long-life cycles and slow growth of most of the seagrasses that impede manipulative experiments and trans-generation assessments^79^. Further space-for-time substitutions to predict functional traits changes due to global warming in seagrasses are necessary. Future trait gradients analysis should consider wider thermal range to sharpen our prediction and establish how closely the highest mean temperature used in the model stands are to the tolerance limit of the seagrass.

## Methods

### Study locations and design

**Figure 6:**
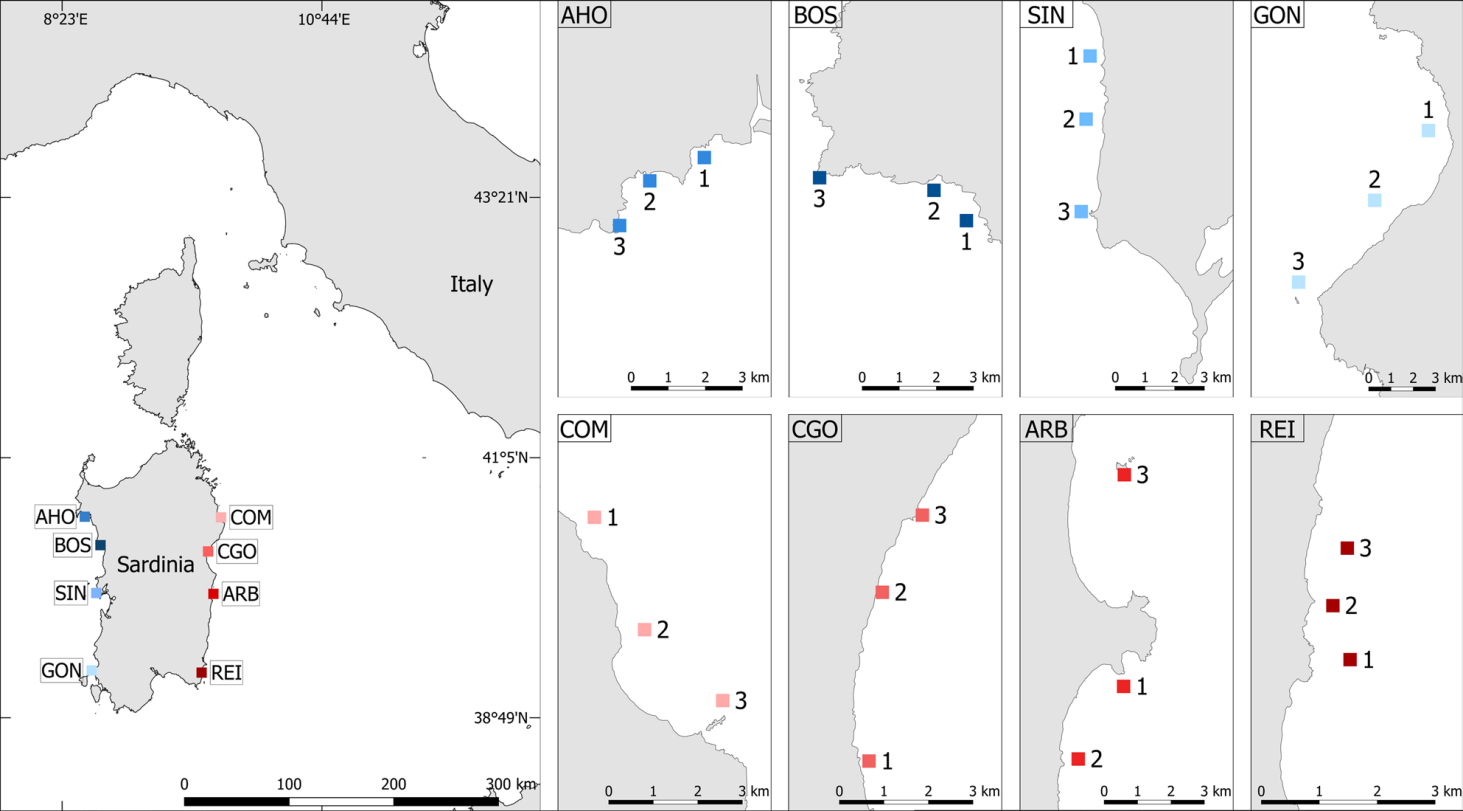
Study locations and areas along the Sardinian coasts. Left-hand map shows locations on the west (in blue) and east (in red) coasts: *AHO *Alghero, *BOS* Bosa, *SIN* Penisola del Sinis, *GON* Gonnesa, *COM* Capo Comino, *CGO* Cala Gonone, *ARB* Arbatax, *REI* Costa Rei. Right-hand inset maps show location of each study area within each location. Map produced with QGIS 3.16 software.

This study was done on the western and eastern coasts of Sardinia (Italy, western Mediterranean Sea, Fig. 6) where differences in water conditions are evident. The western coastline receives Atlantic waters directly through the Western Mid-Mediterranean Current and is also influenced by coastal upwellings^80^. In contrast, the eastern coast is affected by the warm Algerian Current^81^.

Seagrass meadows unaffected from local anthropogenic disturbances (e.g. harbour, fish farming, and urbanisation) were sampled in eight different locations (Fig. 6), with a hierarchical design: for both coasts of Sardinia, four locations were selected (Alghero = AHO, Bosa = BOS, Penisola del Sinis = SIN, and Gonnesa = GON for the west and Capo Comino = COM, Cala Gonone = CGO, Arbatax = ARB, and Costa Rei = REI for the east) from 40°34' to 39°15'N. At each location, three areas 100 s of m apart were randomly selected and sampled at a depth of 10 m.

### Environmental data

**Figure 7:**
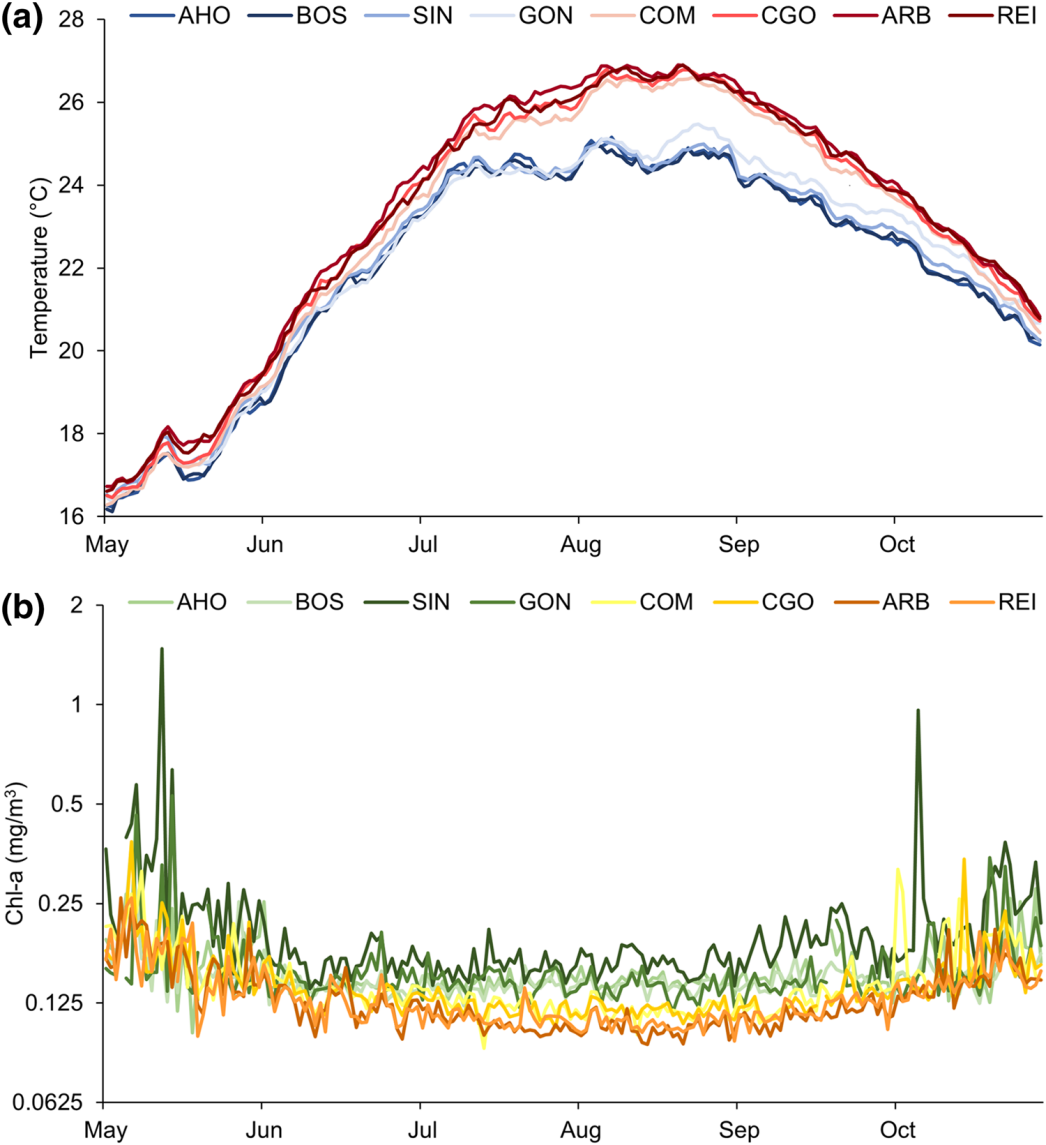
Mean variation from 1st May to 31st October in **(a)** temperature (°C), in blue the western locations and in red the eastern, and **(b)** Chl-*a* (mg/m^3^), in green the western locations and in yellow the eastern). The y axis of the latter plot is log_2_ scale.

For each location the SST for the years 2010–2019 were obtained by the Group for High Resolution Sea Surface Temperature (GHRSST) daily, 1 km resolution SST (G1SST) dataset produced by JPL NASA (https://coastwatch.pfeg.noaa.gov/erddap/griddap/jplMURSST41.html) as a proxy of 10 m subtidal temperature^82^. Moreover, 1 Day Composite, 4 km resolution Chlorophyll-a data from NASA's Aqua Spacecraft (https://coastwatch.pfeg.noaa.gov/erddap/griddap/erdMH1chla1day.html) were extracted for the same years. For the warm season 1st May–31st October (the period of the largest differences between the two coasts), daily SST and Chl-*a* data were averaged through years (Fig. 7) and the mean, maximum and variance for both variables were calculated (Table 4).

**Table 4:** Mean (± SE) temperature and Chl-*a* explanatory variables from May 1st-October 31st (2010–2019).

Location	Temperature (°C)						Chl-*a* (mg/m^3^)					
	Mean ± SE		Max ± SE		Variance ± SE		Mean ± SE		Max ± SE		Variance ± SE	
**West coast**												
AHO	22.09	± 0.14	26.51	± 0.28	7.89	± 0.62	0.149	± 0.001	0.282	± 0.026	0.0009	± 0.0002
BOS	22.06	± 0.15	26.46	± 0.26	7.69	± 0.59	0.144	± 0.001	0.229	± 0.016	0.0005	± 0.0001
SIN	22.23	± 0.15	26.43	± 0.20	7.48	± 0.58	0.184	± 0.004	0.626	± 0.131	0.0073	± 0.0031
GON	22.32	± 0.16	26.44	± 0.27	7.91	± 0.66	0.149	± 0.003	0.358	± 0.038	0.0019	± 0.0005
**East coast**												
COM	22.95	± 0.15	27.43	± 0.30	10.39	± 0.64	0.136	± 0.004	0.346	± 0.096	0.0031	± 0.0022
CGO	23.16	± 0.14	27.7	± 0.27	10.4	± 0.62	0.13	± 0.003	0.302	± 0.074	0.0016	± 0.0010
ARB	23.38	± 0.13	27.76	± 0.27	10.28	± 0.63	0.118	± 0.003	0.201	± 0.026	0.0008	± 0.0003
REI	23.24	± 0.14	27.55	± 0.25	9.98	± 0.52	0.121	± 0.003	0.207	± 0.025	0.0007	± 0.0003

### Seagrass data collection

From 20th June to the 10th July 2020 the density of *Posidonia oceanica* shoots was estimated using 40 × 40 cm quadrats haphazardly placed within meadows (n = 4) and 20 orthotropic shoots were collected at each area. A total of 480 shoots were collected, transported to the laboratory and stored frozen. Sampling was non-lethal and followed the guidelines approved by the Marine Strategy Framework Directive (EC 2008) for the monitoring program. *P. oceanica* shoots were deposited as voucher specimens at the University of Sassari Herbarium (SS, collection 2000/, ID number: SS#14159-SS#14166).

The leaf length, leaf width, number of leaves and necrotic leaf portion were measured following^83^ to estimate *P. oceanica* shoot morphometry. Furthermore, the age reconstruction technique based on the cyclic annual variation of the sheath thickness^84^ was used to estimate shoot productivity through years: therefore, the number of leaves (by counting the scales), rhizome elongation, rhizome width and biomass per year were measured on each shoot (after drying rhizomes for 48 h at 60 °C).

### Data analysis

For each *P. oceanica* variable (shoot density, leaf length, leaf width, number of leaves, necrotic leaf portion, number of scales, rhizome elongation, rhizome width and rhizome biomass) a three-way anova was run to test the effect of ‘Coast’ (C, west vs east), ‘Location’ (L, 4 levels) random nested in C, and ‘Area’ (3 levels) random nested in L. Cochran’s test was used to test variance homogeneity.

With the aim of finding a relationship between the *P. oceanica* and the explanatory variables (mean temperature, maximum temperature, temperature variance, mean Chl-a, maximum Chl-a and Chl-a variance, Table 4), we ran separate multiple linear regression models for each *P. oceanica* response variables. No linear regression was run on leaf length since it is largely affected by herbivore pressure, and it cannot be evaluated unless controlled experiments are performed^85^. Data exploration followed^86^: outliers were inspected with Cleveland dotplots (and removed in four cases) and normality with histograms and Q–Q plots. Rhizome biomass was square root transformed. Collinearity between continuous explanatory variables was inspected with pair-plots, and variance inflation factors (VIFs) were calculated. Several significant correlations were found, particularly, mean temperature, maximum temperature and temperature variance were correlated to each other, as well as mean Chl-a, maximum Chl-a and Chl-a variance. Thus, only mean temperature and mean Chl-a (the variables with VIFs < 3) and their interaction were considered in the analyses, even though the results obtained for each of them can be extended to all the correlated descriptors.

The explanatory variables used in the final model were chosen with a backward selection process^80^. Model validation was run calculating and plotting: (I) standardized residuals against fitted values to assess homogeneity; (II) histogram of the residuals to verify normality; (III) residuals against each explanatory variable that was used in the model; (IV) residuals against each explanatory variable not used in the model. At the end, the model was assessed for influential observations using the Cook distance function.

Correlations between *P. oceanica* shoot density and all the other plant variables were explored at the scale of area to identify eventual plant traits that might derive from a compensatory performance of the plant to temperature and Chl-a. Thus, following the same methodological approach, another multiple linear regression was run to identify the relationship between shoot density and the other response variables. Since rhizome width was correlated to leaf width and rhizome elongation was correlated to rhizome biomass, the model was run using leaf width, number of leaves and scales and rhizome biomass as predictors. All the analyses were run in R Core Team^87^, using the package MASS^88^.
